# Lo mejor del 2020: estudios que marcarán nuestra práctica clínica

**DOI:** 10.47487/apcyccv.v1i4.101.

**Published:** 2020-12-31

**Authors:** Cynthia Paredes-Paucar, Manuel Chacón-Diaz

**Affiliations:** 1 Editor asociado APCyCCV. Cardiología clínica. Instituto Nacional de Cardiología Ignacio Chávez. México DF, México. Cardiología clínica Instituto Nacional de Cardiología Ignacio Chávez México DF México; 2 Editor General APCyCCV. Cardiología clínica. Instituto Nacional Cardiovascular INCOR. Lima, Perú. Cardiología clínica Instituto Nacional Cardiovascular INCOR Lima Perú

“En el medio de la dificultad, yace la oportunidad - Albert Einstein”.

En diciembre de 2019, el síndrome respiratorio agudo severo, coronavirus 2 (SARS-CoV-2) causó un brote de neumonía (COVID-19) que ha llevado a un estado de pandemia hasta la actualidad ^1^. Esto ha generado cambios en todos los aspectos de nuestra vida, y pese a que las muertes por esta pandemia ascienden los 1,7 millones de personas para el año 2020 ^2^, debemos recordar que las muertes por causa cardiovascular (CV) ascienden a 17,9 millones de casos cada año ^3^, siendo esta otra pandemia desatendida principalmente en países de mediano y bajos ingreso, como el nuestro ^3^^,^^4^.

En vista de lo anterior, y pese a las barreras existentes, los avances científicos se continuaron dando y fueron presentados por vía virtual en las reuniones científicas más importantes a nivel mundial. A continuación, resaltamos aquellos que consideramos cambiarán las guías de práctica clínica sobre todo en el área de la insuficiencia cardiaca (IC) y la cardiopatía isquémica.

## Insuficiencia cardiaca

**Figura 1:**
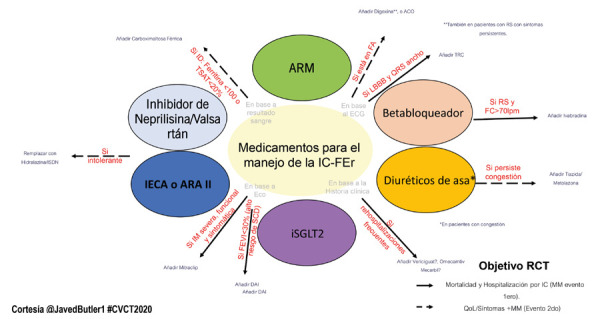
Propuesta de tratamiento en IC-FEr.

El estudio **EMPEROR-Reduced**^5^ encontró que el inhibidor del cotransportador sodio -glucosa 2 (iSGLT2): empaglifozina disminuyó el combinado de muerte CV o primera hospitalización por IC en pacientes con fracción de eyección (FEVI) ≤40%, con un número necesario a tratar (NNT) de 19 a 16 meses, y un efecto significativo desde el día 12 de iniciado el medicamento ^6^. Asimismo, con un efecto añadido de nefro protección (menor tasa de disminución en la función renal, con ↓ en riesgo relativo [RR] de 50%), hace que los iSGLT2 se posicionen con un beneficio de clase, que anticipa sean un pilar de tratamiento de los pacientes con IC con fracción de eyección reducida (IC-FEr) para la próxima guía de IC de la Sociedad Europea de Cardiología 2021 **(**Figura 1**)**.

IC-FEr: insuficiencia cardiaca con fracción de eyección reducida. ECG: electrocardiograma. Eco: ecocardiograma. TSAT: saturación de transferrina. ARM: antagonista de receptor de mineralcorticoide. IECA: inhibidor de la enzima de conversión de angiotensina. ARA II: antagonistas del receptor de la angiotensina II. iSGLT2: inhibidor de receptor del cotransportador sodio-glucosa 2. IM: insuficiencia mitral. FEVI: fracción de eyección de ventrículo izquierdo. SCD: muerte súbita. DAI: desfibrilador automático implantable. FC: frecuencia cardiaca. LBBB: bloqueo de rama izquierda. FA: fibrilación auricular. ACO: anticoagulación oral. TRC: terapia de resincronización. RS: ritmo sinusal. IC: insuficiencia cardiaca. MM: mortalidad y morbilidad. QoL: calidad de vida.

El estudio **VICTORIA**^7^ demostró el beneficio de vericiguat, un estimulador de la guanilato ciclasa soluble, en una población de pacientes con IC con FEVI <45%, sintomática, con hospitalización reciente, y de mayor riesgo por sus características basales (NT-proBNP ≈2816 pg/ml, NYHA III-IV≈41%); logrando la disminución en el evento primario combinado de muerte CV o primera hospitalización por IC (↓riesgo absoluto (RA): 4,2%, NNT=24) en un seguimiento a ~10,8 meses. Por otro lado, **GALACTIC**^8^ puso a prueba el beneficio de omecamtiv mecarbil, un activador selectivo de la miosina cardiaca, en un grupo de pacientes con FEVI ≤35% sintomática y hospitalización reciente en < 1 año, logrando una disminución en visita ambulatoria o a urgencias o de hospitalización por empeoramiento de IC o muerte CV en un 8% (↓RA:2,1, NNT=47) en el seguimiento a 21,8 meses.

Los beneficios añadidos de los iSGLT2 fueron demostrados en el estudio **DAPA-CKD**^9^, donde la dapaglifozina (10 mg) en una población con tasa de filtración glomerular (TFG) de 25-75 mL/min/1,73m^2^ y albuminuria, logró disminuir el evento primario compuesto de disminución sostenida y mayor a 50% en la filtración glomerular o desarrollo de enfermedad renal terminal o muerte de causas renal o CV, con una ↓ de RR en 39%, NNT=19 a un seguimiento de 2,4 años (≈97% de la población con dosis máxima tolerada de IECAS/ARA II en cada brazo). Por lo antes comentado el uso de iSGLT2 se ha ampliado a una población no diabética con IC y enfermedad renal crónica (ERC).

Con un efecto similar, la Sotaglifozina, droga que actúa como iSGLT2 y iSGLT1 en pacientes con diabetes *mellitus* (DM) tipo 1 (inhibe la absorción de glucosa a nivel del túbulo renal proximal y a nivel intestinal) amplia su campo de estudio en **SOLOIST-WHF**^10^ y **SCORED**^11^^)^ que pese a haber sido detenidos por la pandemia, demostraron su eficacia en población con DM2. **SOLOIST-WHF**^10^ aleatorizó a una población hospitalizada por IC, estables o a menos de tres días de su egreso por IC, con NT-proBNP≥600 (≥1800 si fibrilación auricular) y DM2, al uso de sotaglifozina 200 mg (con un incremento a 400 mg si no efectos adversos) vs placebo. El beneficio se logró en una media de 9 meses en el evento primario compuesto de muerte CV y hospitalización por IC y visitas a urgencia por IC con una ↓ RA de 25 eventos por 100 pacientes/año, y un NNT=4 pacientes/año, con una respuesta beneficiosa significativa desde el día 28 de iniciado el medicamento; los efectos adversos más frecuentes encontrados fueron diarrea e hipoglicemia. **SCORED**^11^ estudió a una población con DM2, ERC (TFG: 25-60 mL/min/1,73m^2^) y factor de riesgo CV, logrando el mismo beneficio en evento primario compuesto que SOLOIST-WHF en un seguimiento a 16 meses con una ↓ RA de 1,9%, NNT=54, beneficio también observado tempranamente (tres meses). Además, dentro de los objetivos secundarios se obtiene un beneficio significativo en infarto de miocardio (IM) y evento cerebrovascular (EVC) tanto fatal como no fatal, llevando a la hipótesis del posible efecto antiisquémico de los iSGLT1. Aún queda pendiente la aprobación por la FDA de sotaglifozina para los pacientes con DM2.

En el campo de la IC con fracción de eyección preservada (IC-FEp) y con rango medio (IC-FEm) no existe, a la fecha, medicación que demuestre mejora sostenida en la morbimortalidad, mucho de esto tiene relación por la heterogeneidad de etiologías/fenotipos en este grupo de pacientes. El estudio **PARALLAX**^12^ demostró el potencial beneficio de sacubitril/valsartán sobre la terapia médica individualizada (inhibidores de la renina angiotensina: enalapril, valsartán o ninguno) con resultados favorables en uno de los dos eventos primarios: disminución de NT-proBNP a 12 semanas, pero sin efecto en la prueba de caminata de seis minutos. En los hallazgos exploratorios se observó menor disminución de la función renal y hospitalizaciones por IC a seis meses. Este estudio junto con el estudio PARAGON-HF ^13^, demuestra el potencial efecto beneficioso de sacubitril/valsartán sobre la terapia con otros Inhibidores de renina angiotensina aldosterona (SRAA) en este grupo de pacientes. En base a estos hallazgos y valiéndose de los resultados limítrofes encontrados en el estudio PARAGON-HF, la FDA aprobó este año el uso de sacubitril/valsartán en pacientes con FEVI≥ 45% ^14^.

Independientemente de la presencia o no de anemia, la deficiencia de hierro (DH) se presenta con una prevalencia del 50% en pacientes ambulatorios con IC ^15^. El estudio **AFFIRM-AHF**^16^^)^ demostró la eficacia y seguridad del uso de carboximaltosa férrica intravenosa en pacientes con hospitalización por IC una vez estabilizado el cuadro, que además tenga DH (ferritina<100 ng/mL o ferritina 100-200 ng/mL con saturación de transferrina <20%) y FEVI <50%. El beneficio en hospitalizaciones por IC y muerte CV tuvo un resultado limítrofe con un RR de 0,79 (IC 0,62-1,01, p=0,059). Adjudicándose un efecto relacionado a la pandemia por SARS-CoV-2, se realizó un análisis de sensibilidad pre-COVID-19, donde el poder del estudio fue reanalizado, dando resultados beneficiosos con una ↓ RR de 25%, sobre todo por la disminución del número total de hospitalizaciones por IC.

## Cardiopatía isquémica

**Figura 2:**
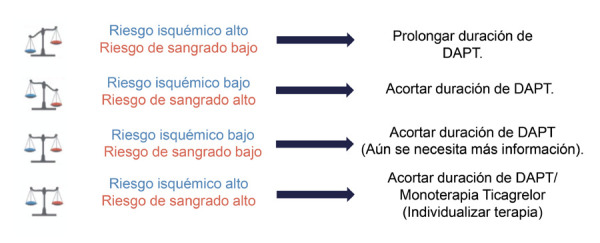
La terapia correcta para el paciente correcto, una sola propuesta no es para todos. DAPT: doble antiagregación.

En base a los estudios previos de desescalación de la terapia con doble antiagregación (DAPT): STOPDAPT-2, SMART-CHOICE, GLOBAL LEADERS, TWILIGHT, se habla cada vez más de una terapia dinámica e individualizada al riesgo isquémico y hemorrágico que presentan los pacientes que van a intervención coronaria percutánea (ICP) tanto en el contexto de síndrome coronario crónico, como en el grupo de pacientes con síndrome coronario agudo (SICA) **(**Figura 2**).**

Los subanálisis del estudio **TWILIGHT** ^(17, 18)^ (con el sesgo, y la falta de poder estadístico propia de los subanálisis), confirmaron el beneficio de ticagrelor como monoterapia después de los tres meses de la colocación de un *stent* liberador de droga (DES) sobre la terapia con aspirina más ticagrelor a doce meses de seguimiento. Los subanálisis incluyeron la evaluación de esta estrategia en pacientes con ICP compleja (definido como 1 o más de los siguientes: tres vasos tratados; ≥3 lesiones tratadas; longitud del *stent* >60 mm (52% de los casos); lesiones en bifurcación tratadas con dos *stent*; *stent* al tronco de la coronaria izquierda o proximal de la descendente anterior; lesión calcificada tratada con aterectomía; *stent* en injerto arterial o venoso o tratamiento de lesión por oclusión total crónica) con beneficio en el evento primario de sangrado BARC tipo 2,3,5 (↓RR 26%, p<0,05) a doce meses a favor de la monoterapia con ticagrelor. El subanálisis de TWILIGHT en la población con DM2 confirmó el mismo beneficio en el evento primario (↓RR 35%, p<0.05); sin incremento significativo en ambos subanálisis en eventos isquémicos con respecto a la terapia control de aspirina y ticagrelor a doce meses. Beneficios similares fueron observados en TICO ^19^**,** que englobó solo pacientes con SICA (36% infarto con elevación de ST), donde el beneficio en el evento primario (sangrado mayor TIMI o evento mayor cardiaco o cerebral) de la monoterapia de ticagrelor a tres meses de la ICP fue principalmente por menores eventos de sangrado mayor (1,7% eventos en terapia con ticagrelor vs 3% en terapia DAPT) a doce meses de seguimiento.

De esto se concluye que la terapia corta con DAPT, seguida de monoterapia con un inhibidor de la P2Y_12_ es una opción en una población de riesgo intermedio, no estaría establecida aún en las guías en aquellos con bajo riesgo de sangrado o alto riesgo isquémico.

El estudio aleatorizado, doble ciego, placebo-controlado: **COLCOT**^20^^)^ demostró en el año 2019 el beneficio de colchicina 0,5 mg/día en una población pos-IM (menor a 30 días) con revascularización completa percutánea. El beneficio fue en la disminución de muerte CV, recuperado de arresto cardiaco, IM, EVC, hospitalización urgente por angina que lleva a la revascularización coronaria, con una ↓ RR 23% en un seguimiento a dos años (principalmente por menor tasa de hospitalización por angina que requiere revascularización). Este año, los subanálisis del COLCOT demostraron que el beneficio mayor se da cuando se inicia la colchicina en los tres primeros días del IM, con una ↓ RR 48%, p<0,05 en el evento primario antes mencionado, a diferencia de iniciarlo días después del IM ^21^. Asimismo, en el subanálisis en población diabética se observó una ↓ RR 35%, p<0,05 en el evento primario ^22^. Los eventos adversos como la neumonía (p<0,05) fueron más frecuentes en el grupo de pacientes con colchicina siendo la posible justificación para el aumento numérico no significativo de muerte por causas no CV.

El estudio **LoDoCo2**^23^^)^ apoyó el beneficio de la colchicina, esta vez en una población con síndrome coronario crónico con una condición estable por lo menos de seis meses antes de su aleatorización, con un beneficio de dosis baja de colchicina en el evento primario compuesto de muerte CV, IM espontáneo, EVC isquémico, revascularización coronaria por isquemia, con una ↓ RA 1,1%, p<0,001 a 28,6 meses. El beneficio se dió a favor de menor revascularización e IM. El incremento en la tasa de muerte por causa no CV obliga a evaluar cual es la población con mayor beneficio y menor riesgo para el uso de esta droga.

Dentro de otros aspectos de la prevención secundaria en la cardiopatía isquémica, el uso de ácido eicosapentaenoico altamente purificado (etil icosapent) ha demostrado efectos pleiotrópicos, más allá del control de triglicéridos; así, el estudio **EVAPORATE**^25^, estudio aleatorizado, doble ciego y placebo-controlado, demostró el beneficio añadido a la terapia con estatinas del uso de 2 g cada 12 h de ácido etil icosapent en la estabilización de la placa (menor atenuación de placa en 17% evaluado por tomografía coronaria), y con posible reducción de esta en un seguimiento de 18 meses.

Por otro lado, en la terapia transfusional en pacientes con SICA y anemia, existe un dilema y vacío en las guías actuales de práctica clínica ^26^^,^^27^, acerca de esto existe poco o nada de estudios con buen nivel de evidencia científica. El estudio **REALITY**^28^, aleatorizado y abierto, de no inferioridad, planteó contrastar la terapia transfusional restringida (transfusión si la hemoglobina (hb) ≤ 8 g/dL, con una meta en Hb de 8-10 g/dL) vs. una terapia liberal (transfusión si la Hb ≤10 g/dL, con una meta de >11 g/dL) en pacientes con SICA, excluyendo aquellos con choque, sangrado masivo o que amenace la vida, IM por ICP o cirugía de revascularización, transfusión previa en <30 días o desorden hematológico conocido. Con respecto al objetivo primario combinado a 30 días de muerte, reinfarto, EVC y revascularización urgente mediado por isquemia, no se vio diferencias significativas (HR 0,77, 95%IC: 0,5-1,18, p<0,05 para no inferioridad). Asimismo, la estrategia restrictiva resultó ser más costo-efectiva, con menores efectos adversos con una diferencia significativa en menor tasa de infecciones y lesión pulmonar con respecto a la estrategia liberal. Actualmente esta en curso el estudio MINT ^29^ que busca validar la misma estrategia de transfusión, pero un estudio diseñado con efectos en superioridad.

## Misceláneos

En el campo de las cardiomiopatías, el avance más importante ha sido el publicado en el estudio de fase 3 **EXPLORER-HCM**^30^^)^ en la población con cardiomiopatía hipertrófica obstructiva (CMHO). Este estudio aleatorizado, doble ciego, placebo controlado, demostró el beneficio en términos funcionales de mavacamtem, primero en su clase como inhibidor de la miosina cardiaca, en pacientes con CMHO con gradiente en tracto de salida (GTS) > 50 mmHg, sintomáticos (NYHA II-III) añadido a la terapia habitual con betabloqueantes o calcio antagonistas. El objetivo primario fue el aumento en el consumo pico de oxigeno de al menos 1,5 mL/kg asociada a mejoría de al menos una clase funcional de la NYHA o como un aumento en el consumo pico de O^2^ de al menos 3,0 mL/min sin deterioro en la clase funcional. Mavacamtem logró este efecto en un 37% vs. un 17% en la población placebo (diferencia + 19,4%; p= 0, 0005) a un seguimiento de 30 semanas. Asimismo, los pacientes en el grupo intervención lograron disminuir el GTS posejercicio en 36 mmhg, con una diferencia significativa. Si bien los resultados no son en eventos clínicos duros como mortalidad o en hospitalizaciones, es sin duda un estudio que abre la puerta a una terapia médica especifica no existente en este grupo de pacientes.

Con respecto a las valvulopatías, los estudios más relevantes han sido en cuanto al uso de terapia antitrombótica en el implante percutáneo de válvula aortica (TAVI) y válvula bioprotésica mitral. El estudio **POPular TAVI**, aleatorizó pacientes posimplante de TAVI, que no hayan recibido un DES en ≤ 3meses o *stent* sin droga (BMS) en ≤1 mes. La Cohorte B ^(^^31^, agrupo pacientes con requerimiento de terapia con anticoagulación (~95% de los casos por FA), en un estudio aleatorizado, prospectivo y abierto, donde demostró el riesgo de añadir clopidogrel a la terapia de anticoagulación (OAC) (~20% anticoagulante directo) por tres meses, posterior al implante de TAVI con un ↓ RR de 37% de todo sangrado y de 36% en sangrado no relacionado al procedimiento, ambos con diferencia significativa a doce meses con respecto al grupo control (OAC). El aumento de riesgo en el grupo con clopidogrel fue a expensas de sangrado menor, relacionados a la zona de punción (equivalente a BARC 2 o 3a). Por otro lado, el estudio POPular TAVI Cohorte A ^32^, en una misma población que la anterior, pero sin requerimiento de terapia con anticoagulación; demostró que la monoterapia con ácido acetilsalicílico (AAS) fue no inferior a la terapia doble (AAS y clopidogrel por tres meses pos-TAVI) para disminuir los eventos isquémicos a expensas de menor sangrado en un seguimiento a doce meses.

Asimismo, la terapia con TAVI, buscó ampliar su indicación a pacientes con estenosis aórtica severa por válvula aortica bicúspide de bajo riesgo. El estudio **Evolut low risk bicuspid**^33^, realizado de forma no aleatorizada, prospectiva, en 25 centros en Estados Unidos, demostró la eficacia y seguridad del uso de TAVI con el uso de válvulas autoexpandibles EVOLUT R o EVOLUT PRO, en pacientes con válvula aórtica bicúspide confirmada por tomografía computarizada, con riesgo quirúrgico bajo (STS<3%). En el análisis a 30 días se encontró un 1,3% de muerte por todas las causas y EVC incapacitante y la eficacia del procedimiento (evaluado por la ausencia de mortalidad, correcta posición de la bioprótesis a siete días) llegó a un 95,3%.

Por último, la terapia con anticoagulantes directos en pacientes con fibrilación auricular (FA) y válvula bioprotésica mitral, encontró en el estudio **RIVERS**^34^ (aleatorizado, prospectivo y abierto) la no inferioridad de rivaroxaban 20 mg/día (15 mg/día si TFG 30-49) comparado con warfarina (INR meta 2-3 con un tiempo en rango terapéutico promedio de 65%) en el objetivo primario compuesto de muerte, evento adverso mayor CV o sangrado mayor a doce meses (p<0,0001). Pese a las limitaciones de diseño metodológico, este estudio promete cambiar las recomendaciones en las guías de práctica clínica.

Existen otros estudios que seguramente resaltarán a medida que avancen con más información, como son: EAST-AFNET, RATE-HF, HOME-PE, VENUS, SPYRAL-HTN, RHAPSODY, TAILOR PCI, entre otros. En nuestro país donde la enfermedad cardiovascular se ha mantenido desatendida desde hace años, los efectos perjudiciales de la enfermedad por SARS-CoV-2 tanto por el aislamiento, la infección y la no atención oportuna de enfermedades cardiovasculares se verá plasmado en mayores tasas de morbimortalidad cardiovascular a las ya actualmente reconocidas. Es nuestro rol mantener una actitud científica, aun en este contexto, para seguir brindando información y tratamiento con mejor nivel de evidencia a esta población en ya creciente aumento.

En este contexto, este cuarto número de la revista presenta entre algunos de sus temas, un interesante artículo de controversia, donde dos grupos de cardiólogos ponen su conocimiento y experiencia en el diagnóstico y manejo del síndrome coronario crónico de alto riesgo con el tema: **«¿Debo tratar un síndrome coronario crónico de alto riesgo invasivamente desde el inicio?»,** Ortiz *et al*. plantean el manejo invasivo de inicio asociado al tratamiento médico y Carrasco *et al.* plantean solo el tratamiento médico de inicio, ambos grupos teniendo como base la evidencia sobre el tema. Echeverri *et al*. nos presentan un refrescante artículo de revisión para no pasar por alto **«Patrones electrocardiográficos de alto riesgo en pacientes con síndrome coronario agudo».** Guzmán *et al.* presentan en su artículo original **«Características actuales y factores de riesgo de mortalidad en choque cardiogénico por infarto de miocardio en un hospital latinoamericano»**, la forma de presentación de esta entidad en el INCOR, su elevada mortalidad y el uso de escores de riesgo para estratificarla, además de tener el honor de contar con una brillante editorial a cerca del artículo por parte del profesor Holger Thiele, líder mundial en el estudio del choque cardiogénico.

Los miembros del equipo editorial de Archivos Peruanos de Cardiología y Cirugía Cardiovascular esperamos que los artículos de este número sean de su interés.
